# Diagnostic biomarker candidates for pulpitis revealed by bioinformatics analysis of merged microarray gene expression datasets

**DOI:** 10.1186/s12903-020-01266-5

**Published:** 2020-10-12

**Authors:** Ming Chen, Junkai Zeng, Yeqing Yang, Buling Wu

**Affiliations:** 1 Stomatological Hospital, Southern Medical University, Guangzhou, China; 2 School of Stomatology, Southern Medical University, Guangzhou, China; 3 Nanfang Hospital, Southern Medical University, Guangzhou, China; 4 Shenzhen Stomatology Hospital (Pingshan), Southern Medical University, Shenzhen, Guangdong 510515 P.R. China

**Keywords:** Pulpitis, Bioinformatics analysis, Diagnostic biomarker, Microarray gene expression dataset

## Abstract

**Background:**

Pulpitis is an inflammatory disease, the grade of which is classified according to the level of inflammation. Traditional methods of evaluating the status of dental pulp tissue in clinical practice have limitations. The rapid and accurate diagnosis of pulpitis is essential for determining the appropriate treatment. By integrating different datasets from the Gene Expression Omnibus (GEO) database, we analysed a merged expression matrix of pulpitis, aiming to identify biological pathways and diagnostic biomarkers of pulpitis.

**Methods:**

By integrating two datasets (GSE77459 and GSE92681) in the GEO database using the sva and limma packages of R, differentially expressed genes (DEGs) of pulpitis were identified. Then, the DEGs were analysed to identify biological pathways of dental pulp inflammation with Gene Ontology (GO) analysis, Kyoto Encyclopedia of Genes and Genomes (KEGG) pathway enrichment analysis and Gene Set Enrichment Analysis (GSEA). Protein–protein interaction (PPI) networks and modules were constructed to identify hub genes with the Search Tool for the Retrieval of Interacting Genes/Proteins (STRING) and Cytoscape.

**Results:**

A total of 470 DEGs comprising 394 upregulated and 76 downregulated genes were found in pulpitis tissue. GO analysis revealed that the DEGs were enriched in biological processes related to inflammation, and the enriched pathways in the KEGG pathway analysis were cytokine-cytokine receptor interaction, chemokine signalling pathway and NF-κB signalling pathway. The GSEA results provided further functional annotations, including complement system, IL6/JAK/STAT3 signalling pathway and inflammatory response pathways. According to the degrees of nodes in the PPI network, 10 hub genes were identified, and 8 diagnostic biomarker candidates were screened: PTPRC, CD86, CCL2, IL6, TLR8, MMP9, CXCL8 and ICAM1.

**Conclusions:**

With bioinformatics analysis of merged datasets, biomarker candidates of pulpitis were screened and the findings may be as reference to develop a new method of pulpitis diagnosis.

## Background

Dental pulp is loose connective tissue in the pulp cavity surrounded by rigid dentin and is necessary for tooth nutrition, innervation, and immunocompetency [1]. When the hard dental tissue is broken, various stimuli may induce pathological changes in dental pulp, which is difficult to heal due to the lack of collateral circulation [2]. Pulpitis is an inflammatory disease of the dental pulp, and bacterial infection is considered to be the most important trigger of pulpitis [3]. Whether a pathological change occurs in dental pulp and the degree of lesions are related not only to the virulence and amount of bacteria but also to the defensive capacity of the host [4]. Tertiary dentin is formed reactively when dental pulp is stimulated, and a balance exists between inflammation and reparative processes. If harmful stimuli cannot be removed in time, increasing numbers of immune cells are recruited to the pulp tissue [5] and the immune-inflammatory balance is disrupted. Thus, irreversible pulpitis can occur as a result of uncontrollable inflammation caused by invading bacteria [6]. Without appropriate treatment, pulpitis may result in pulp necrosis, periapical periodontitis and more severe conditions [7].

Different therapies for pulp disease can be selected according to the evaluation of pulp inflammation. Given the importance of tooth preservation, vital pulp therapies such as pulp capping, pulpotomy, and stepwise excavation of caries may be more appropriate than root canal treatment under some circumstances [8]. Currently, guided by the criteria of the American Association of Endodontists (AAE), medical history and clinical examination are the main methods used to evaluate pulp inflammation severity, considering information such as pain quality and history and responses to pulp sensitivity tests [9]. Decisions regarding the preservation or removal of vital pulp depend on whether the pulpitis is considered reversible or irreversible [10]. However, histopathological examinations have revealed weak correlations between clinical features and pulp status [6]. Thus, tissues collected from cases diagnosed as irreversible pulpitis may not present severe inflammation in pathologic examination [11].

In clinical practice, incomplete or ambiguous clinical data make it difficult to determine whether a deep carious lesion has led to the widespread infection of dental pulp (irreversible pulpitis), the infection of only a small amount of tissue near the lesion, or no infection of pulp tissue (reversible pulpitis) [6]. Hence, clinical diagnosis has limitations for determining the degree of pulp inflammation [10]. As histopathological examinations of pulp tissue cannot be completed without tooth extraction, a new method of non-invasive pulp diagnostics is needed.

Dental pulp is not isolated in the oral cavity and releases many biological products to the external environment in response to external harmful stimuli [12–14]. At the cellular or molecular level, a wide range of molecules are released during pulpal and periapical inflammation, including cytokines, proteases, inflammatory mediators, growth factors, and antimicrobial peptides [15, 16]. Measurable levels of molecules can be found not only in pulp tissue but also in pulpal blood [17], dentinal fluid [18, 19], periapical fluid [1], and gingival crevicular fluid [20], which can be collected non-invasively and analysed without extirpating the pulpal tissue [19, 21, 22]. The factors that can be used to assess the level of pulp inflammation are called biomarkers of pulpitis [23]. Belonging to the matrix metalloproteinase (MMP) family, MMP9 can accelerate gelatinolytic activity in inflamed pulp tissue [24]. The levels of MMP9 have been found to be significantly increased in pulp blood collected from pulp exposure [17]. In addition, biomarkers in dental fluid allowing potential diagnosis of pulpitis have been identified [19]. An analysis of gingival crevicular fluid from teeth with irreversible pulpitis showed that the levels of interleukin-8 (CXCL8) were significantly higher than those in healthy contralateral teeth [20]. In addition, the upregulation of active TIMP-2 and myeloperoxidase (MPO) has been observed in inflamed pulp tissue [25]. Employed together with clinical examinations, biomarkers may serve as diagnostic tools to identify different stages of pulpitis [26].

In this study, we focused on gene expression in pulp tissue from pulpitis patients. We selected and analysed two microarray platform datasets in the GEO database, integrated the datasets and identified DEGs between pulpitis and normal pulp tissues. Then, GO enrichment analysis, KEGG pathway analysis and GSEA were used to analyse the major biological functions of the DEGs. Ten hub genes related to pulpitis were identified by constructing a PPI network with Cytoscape. The aim of the present study was to identify candidate biomarkers for pulpitis diagnosis and prognosis based on functional and molecular analyses by evaluating DEGs in pulpitis and normal tissue.

## Methods

Two microarray datasets of pulpitis from GEO database were retrieved, with the keywords: “pulpitis”, “*Homo sapiens*” and “dental pulp”. The diagnostic criteria of normal pulp and pulpitis were in line with endodontics diagnoses system in accordance with the American Association of Endodontists (AAE) guidelines [27]. GSE77459 includes 6 samples of pulpitis and 6 samples of normal pulps, and GSE92681 includes 7 samples of pulpitis and 5 samples of normal pulps, respectively using the microarray platform GPL17692 and GPL16956. The details of both studies are shown in Table 1.

**Table 1:** Summary of two individual datasets of pulpitis

GEO gene set ID	GSE77459	GSE92681
Title	Gene Expression Profile of Pulpitis	Differential Expression of LncRNAs and mRNAs in normal and inflamed human pulp
Platform	GPL17692: Affymetrix Human Gene 2.1 ST Array [transcript (gene) version]	GPL16956: Agilent 045997 Arraystar human lncRNA microarray V3 (Probe Name Version)
Diagnostic criteria	AAE	AAE
Number of normal samples vs. pulpitis sample	6 vs. 6	5 vs. 7
Clinical data		
Normal tissues	Picked from healthy teeth extracted for various reasons	Picked from healthy teeth extracted for various reasons
Pulpitis tissues	Picked from teeth diagnosed with irreversible pulpitis	Picked from teeth diagnosed with irreversible pulpitis
PubMed ID	27,052,691	29,079,059

### Data processing

After removing the probes of lncRNA in GSE92681, mRNAs of two datasets were merged into one file, and then ComBat normalization in SVA package (https://bioconductor.org/packages/sva/) was used to remove batch effects based on the standard protocol [28, 29]. Then, the raw data was converted into the form of an expression matrix and handled with the Linear Models for Microarray data (limma, https://bioconductor.org/packages/limma/) package in Bioconductor. Up- or downregulated DEGs between samples of pulpitis and normal pulps were identified with the cut-off criteria of adjusted *p*-value (adj. P. val) < 0.05 and |fold change (FC)| > 2.

### Functional analysis of DEGs

GO enrichment and KEGG pathway analyses were used to investigate the functional progression of pulpitis. Biological process (BP), molecular function (MF), cellular component (CC) in GO analysis and potential pathways in KEGG analysis were performed in the Database for Annotation Visualization and Integrated Discovery (DAVID) (https://david.ncifcrf.gov/). *P* < 0.05 and false discovery rate (FDR) < 5% were used as the cut-off criteria.

### The gene set enrichment analysis

To further explore the function of DEGs in inflammatory progression, GSEA was performed using h.all.v7.0.symbols.gmt (http://software.broadinstitute.org/gsea/ downloads.jsp) as a reference gene set [30]. The GSEA software (version 4.0) is available on the GSEA website (http://software.broadinstitute.org/gsea/index.jsp). Gene set permutations were performed 1000 times, and the pathway set list was sorted by the Normalized Enrichment Score (NES). *P* < 0.05 and FDR < 0.25 were considered statistically significant.

### PPI network analysis and hub gene identification

The PPI network of DEGs was constructed using the Search Tool for THE Retrieval of Interacting Genes (STRING) database (Version 11.0, http://string-db.org/). PPI pairs and PPI network were visualized in the Cytoscape software (Version 3.7.1), and cytohHubba and MCODE plugin in Cytoscape were used to calculate the degrees of protein nodes and to select the significant modules. Top 10 genes were identified as hub genes.

## Results

### Dataset integration and identification of DEGs

**Fig. 1:**
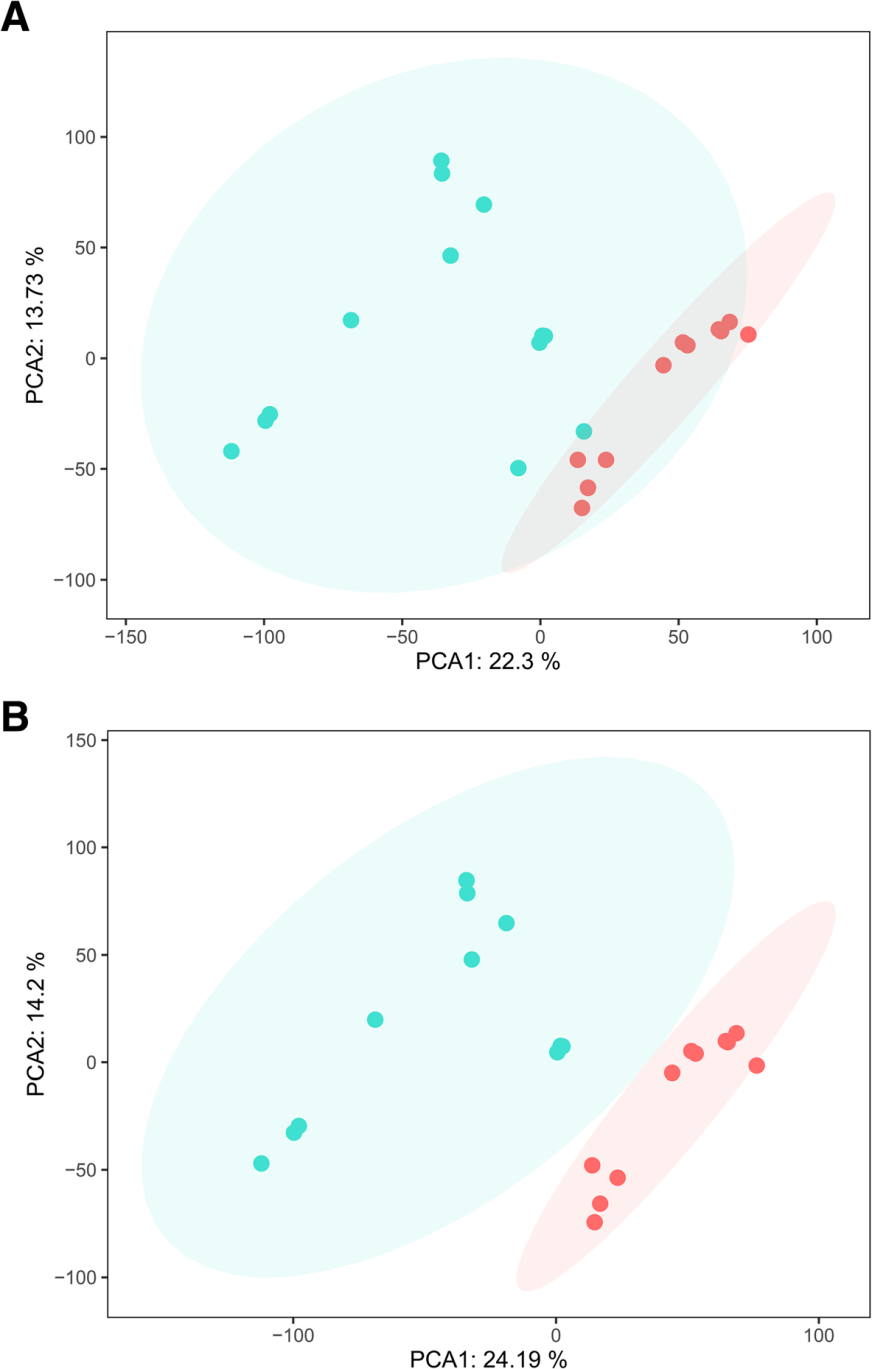
PCA results before and after batch effect removal

**Fig. 2:**
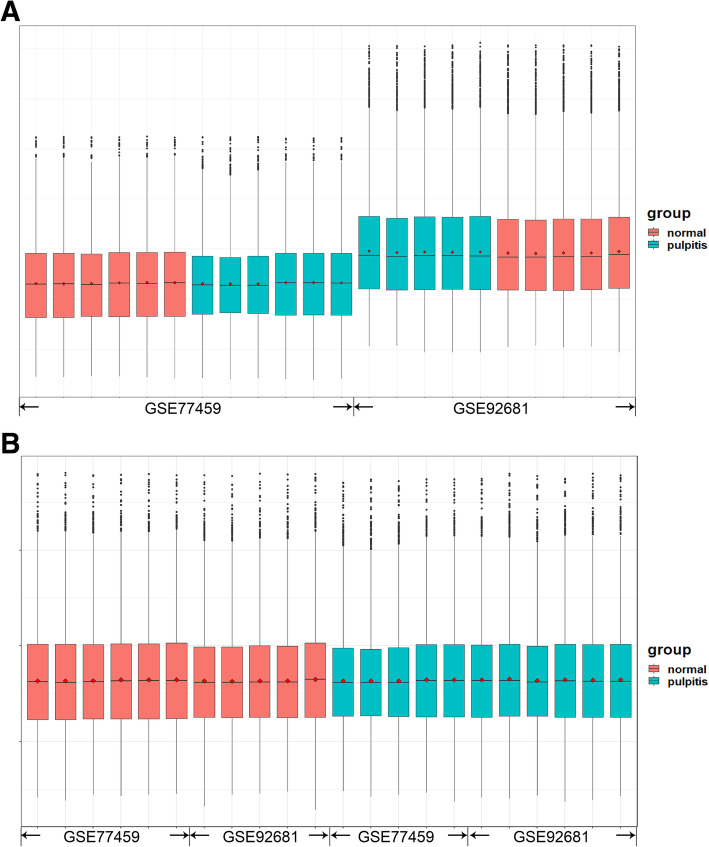
Box plot of data from 22 samples in the pooled dataset that were normalized. The X-axis presents samples from the dataset, and the Y-axis presents normalized intensity values. **a** Box plot showing the expression profiles of the pooled dataset before normalization; **b** Box plot showing the expression profiles of the pooled dataset after normalization using the SVA package

**Fig. 3:**
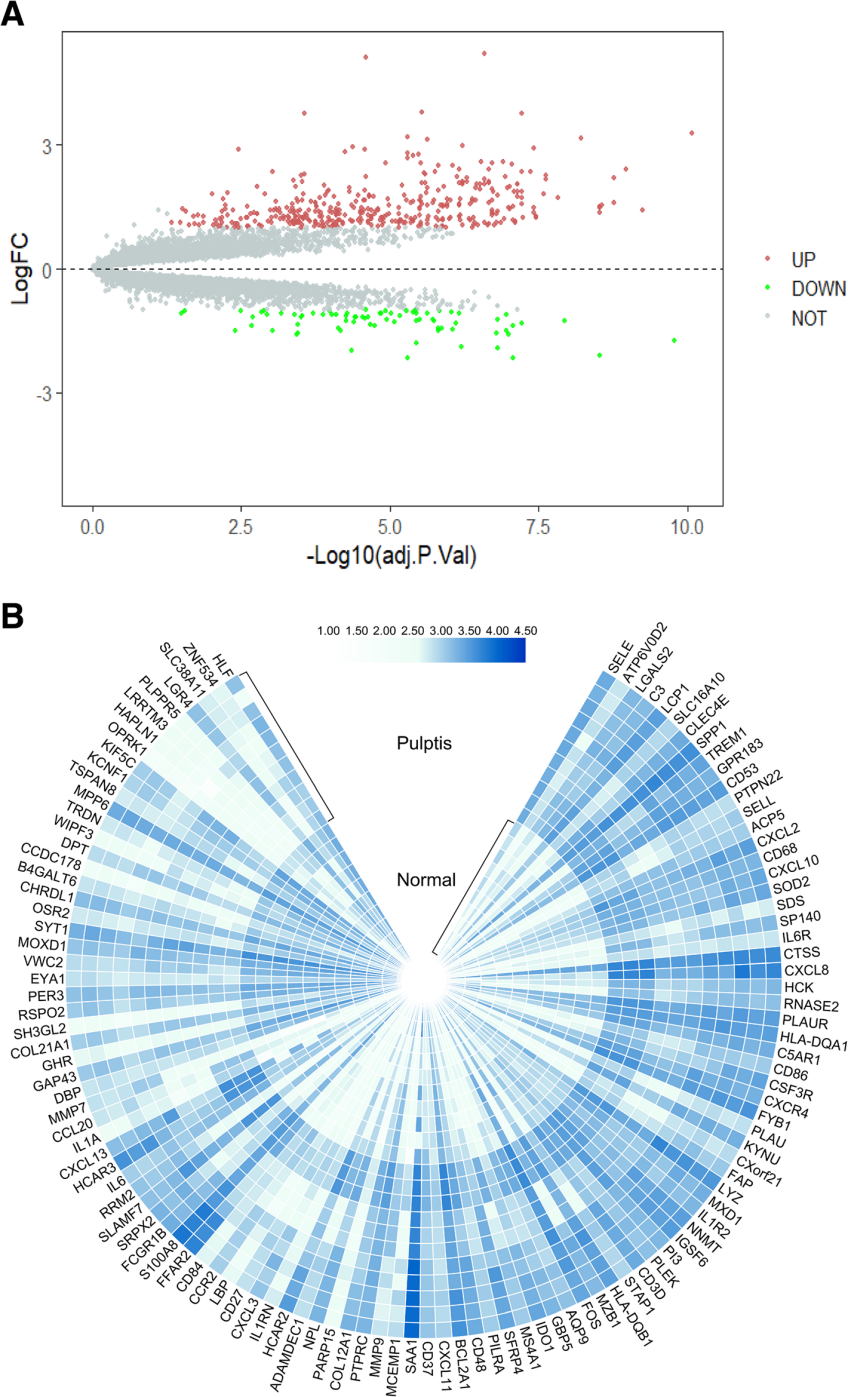
Volcano plot and heatmap of DEGs. **a** Volcano plot for the DEGs in the merged dataset. The X-axis shows the -log10 adj.*P*, and the Y-axis shows the log FC. The DEGs were identified according to the criteria |FC| > 1.0 and adj. P. val < 0.05. The red and green circles denote upregulated genes and downregulated genes in pulpitis, respectively. The grey circles denote genes with no significant difference in expression between pulpitis and healthy tissue. **b** Heatmap of the DEGs in the merged dataset. Blue and white represent upregulation and downregulation, respectively, of the mRNAs

According to the principal component analysis (PCA), the data from two samples, GSM2434473 and GSM2434475, were excluded (Fig. 1). Then, an expression matrix of normal pulp and pulpitis was obtained, representing 12,813 mRNAs and 22 groups (11 normal samples and 11 pulpitis samples, Fig. 2). A total of 470 DEGs were screened from the two merged microarray platform datasets, including 394 upregulated genes and 75 downregulated genes in pulpitis relative to normal tissue, shown in a volcano plot and heatmap (Fig. 3). Details of the expression matrix and DEGs are provided in Additional files 1 and 2.

### Functional analysis of DEGs

**Fig. 4:**
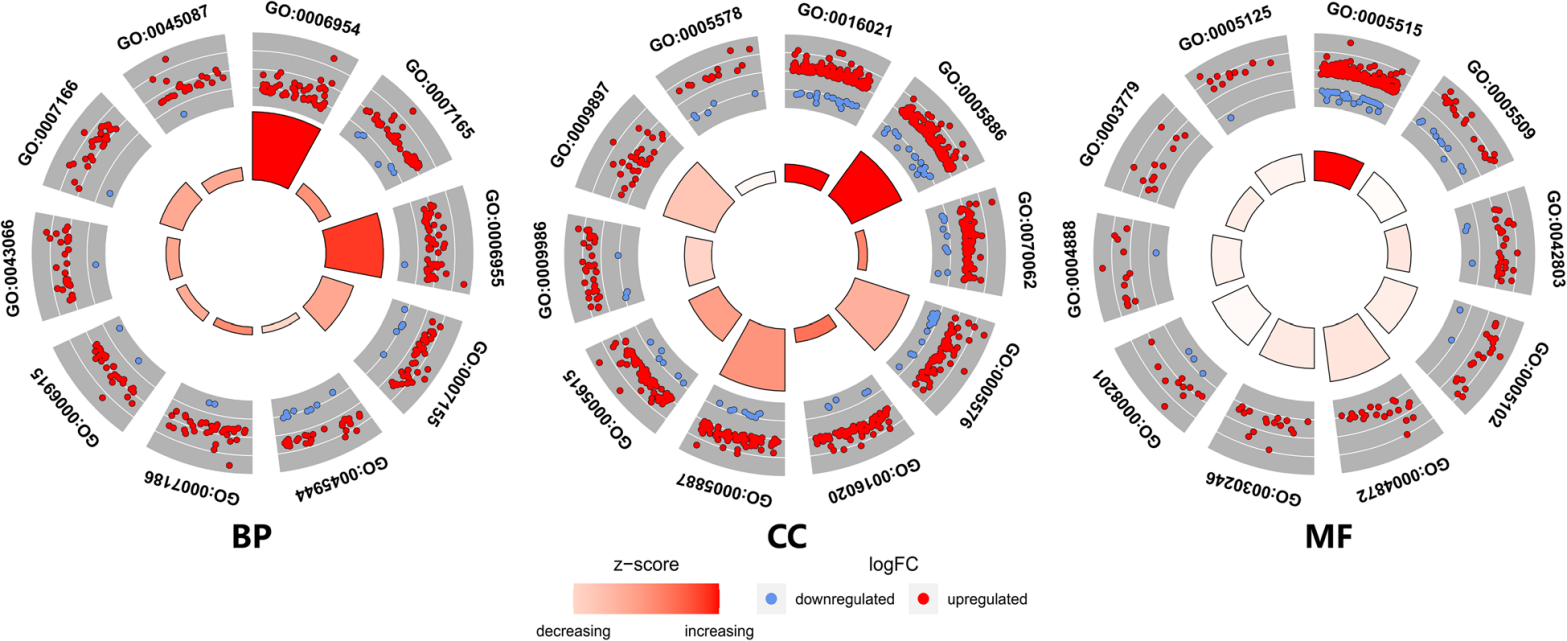
Top 10 terms from the GO enrichment analysis, including BP, MF and CC. Red and dots represent the upregulated genes and downregulated genes respectively. The size and colour of the sectors indicate the adjusted adj. P. val and stand score (z-score), respectively, of each GO term

**Fig. 5:**
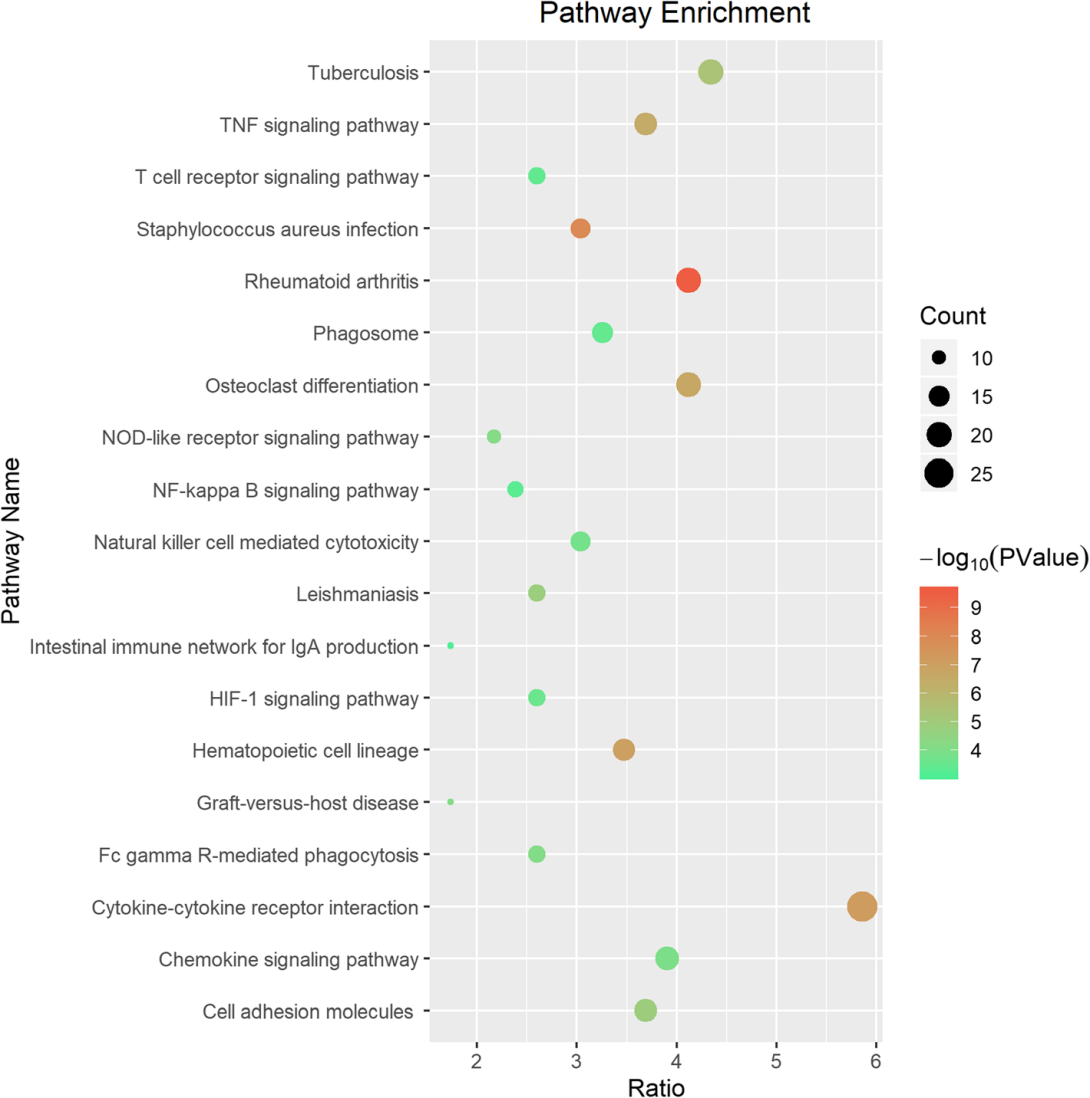
Top 20 pathways from the KEGG pathways analysis. The X-axis presents the ratio of enriched genes to all of the DEGs, and the Y-axis presents the names of the KEGG pathway terms. The size and colour of the circles indicate the number of genes and -log10(*P*-Value), respectively, for each enriched term

GO BP, CC and MF and KEGG pathway analyses were performed for functional analysis of the DEGs. Regarding BP terms, the DEGs were enriched in inflammatory response, signal transduction, immune response and cell adhesion. Regarding CC terms, the DEGs were enriched in integral component of membrane, plasma membrane, and extracellular exosome. Regarding the MF terms, the DEGs were enriched in protein binding, calcium ion binding, and protein homodimerization activity (Fig. 4, Table 2). The significantly enriched KEGG pathways of the DEGs included cytokine-cytokine receptor interaction, osteoclast differentiation, chemokine signalling pathway, NF-κB signalling pathway, T cell receptor signalling pathway and other pathways related to pulp inflammation (Fig. 5). The detailed results of the GO enrichment and KEGG pathway analyses are provided in Additional files 3 and 4.

**Table 2:** Supplementary information of GO analysis

Category	ID	Term	adj. P. val
BP	GO:0006954	inflammatory response	7.13E-24
BP	GO:0007165	signal transduction	3.73E-05
BP	GO:0006955	immune response	3.36E-20
BP	GO:0007155	cell adhesion	7.95E-12
BP	GO:0045944	positive regulation of transcription from RNA polymerase II promoter	0.005548688
BP	GO:0007186	G-protein coupled receptor signalling pathway	0.002323744
BP	GO:0006915	apoptotic process	0.000255676
BP	GO:0043066	negative regulation of apoptotic process	3.66E-05
BP	GO:0007166	cell surface receptor signalling pathway	7.13E-09
BP	GO:0045087	innate immune response	3.69E-05
CC	GO:0016021	integral component of membrane	7.35E-06
CC	GO:0005886	plasma membrane	1.69E-12
CC	GO:0070062	extracellular exosome	0.00028413
CC	GO:0005576	extracellular region	8.34E-13
CC	GO:0016020	membrane	2.96E-05
CC	GO:0005887	integral component of plasma membrane	1.08E-13
CC	GO:0005615	extracellular space	1.57E-08
CC	GO:0009986	cell surface	1.72E-07
CC	GO:0009897	external side of plasma membrane	2.35E-12
CC	GO:0005578	proteinaceous extracellular matrix	0.000108122
MF	GO:0005515	protein binding	0.002283082
MF	GO:0005509	calcium ion binding	0.003507755
MF	GO:0042803	protein homodimerization activity	0.014495214
MF	GO:0005102	receptor binding	0.000515236
MF	GO:0004872	receptor activity	1.96E-06
MF	GO:0030246	carbohydrate binding	0.00011033
MF	GO:0008201	heparin binding	0.000173777
MF	GO:0004888	transmembrane signalling receptor activity	0.002616585
MF	GO:0003779	actin binding	0.043764465
MF	GO:0005125	cytokine activity	0.004406936

### Gene set enrichment analysis

**Fig. 6:**
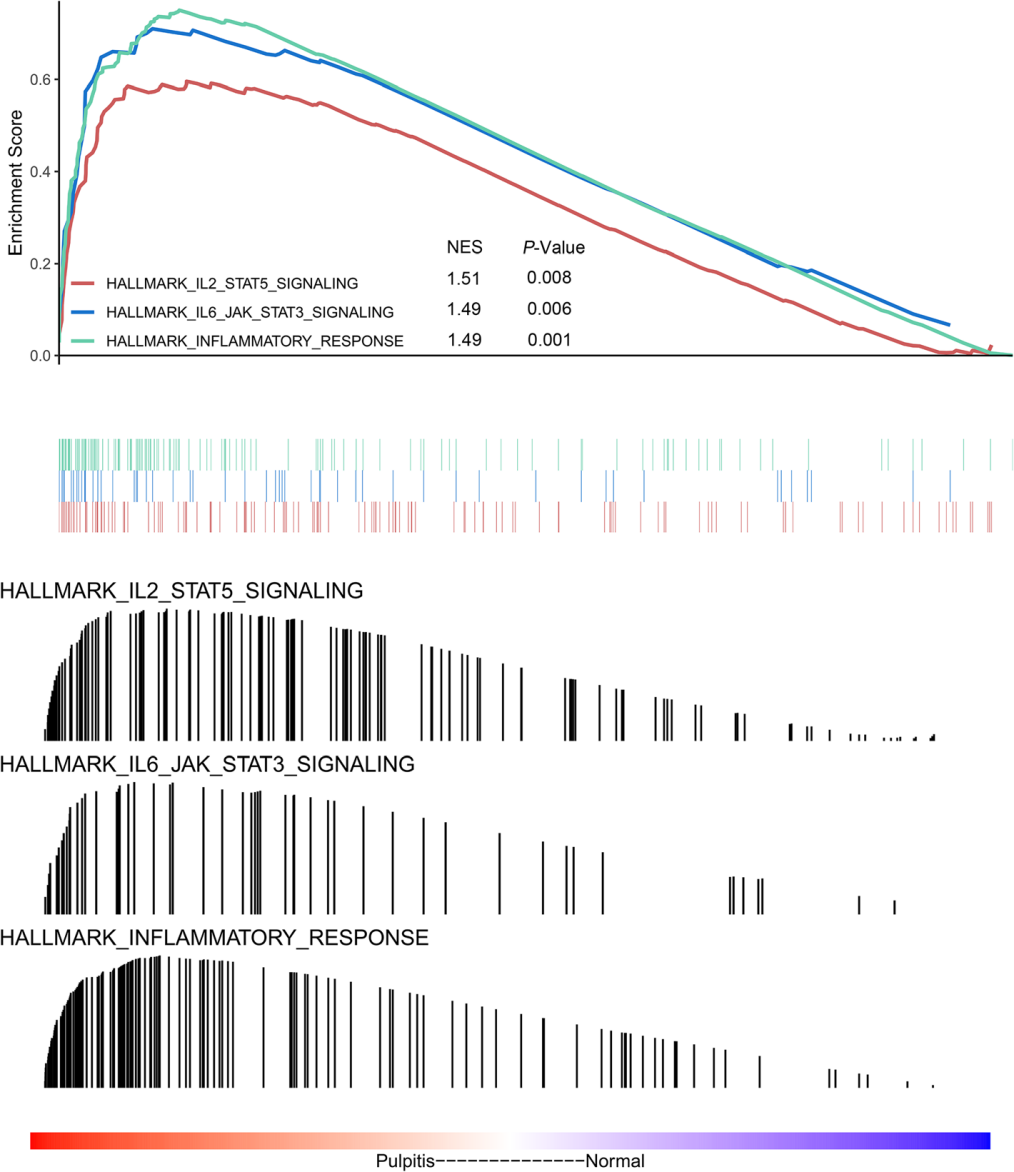
Gene Set Enrichment Analysis results. The red, blue and green lines represent the IL2/STAT5 signalling pathway, IL6/JAK/STAT3 signalling pathway and inflammatory response pathways, respectively. NES: Normalized Enrichment Score

The GSEA results revealed that the enriched biological processes mainly involved the IL2/STAT5 signalling pathway (NES = 1.51; *P* = 0.008), the IL6/JAK/STAT3 signalling pathway (NES = 1.49; *P* = 0.006) and inflammatory response pathways (NES = 1.49; *P* = 0.001), as shown in Fig. 6.

### PPI network and hub genes

**Fig. 7:**
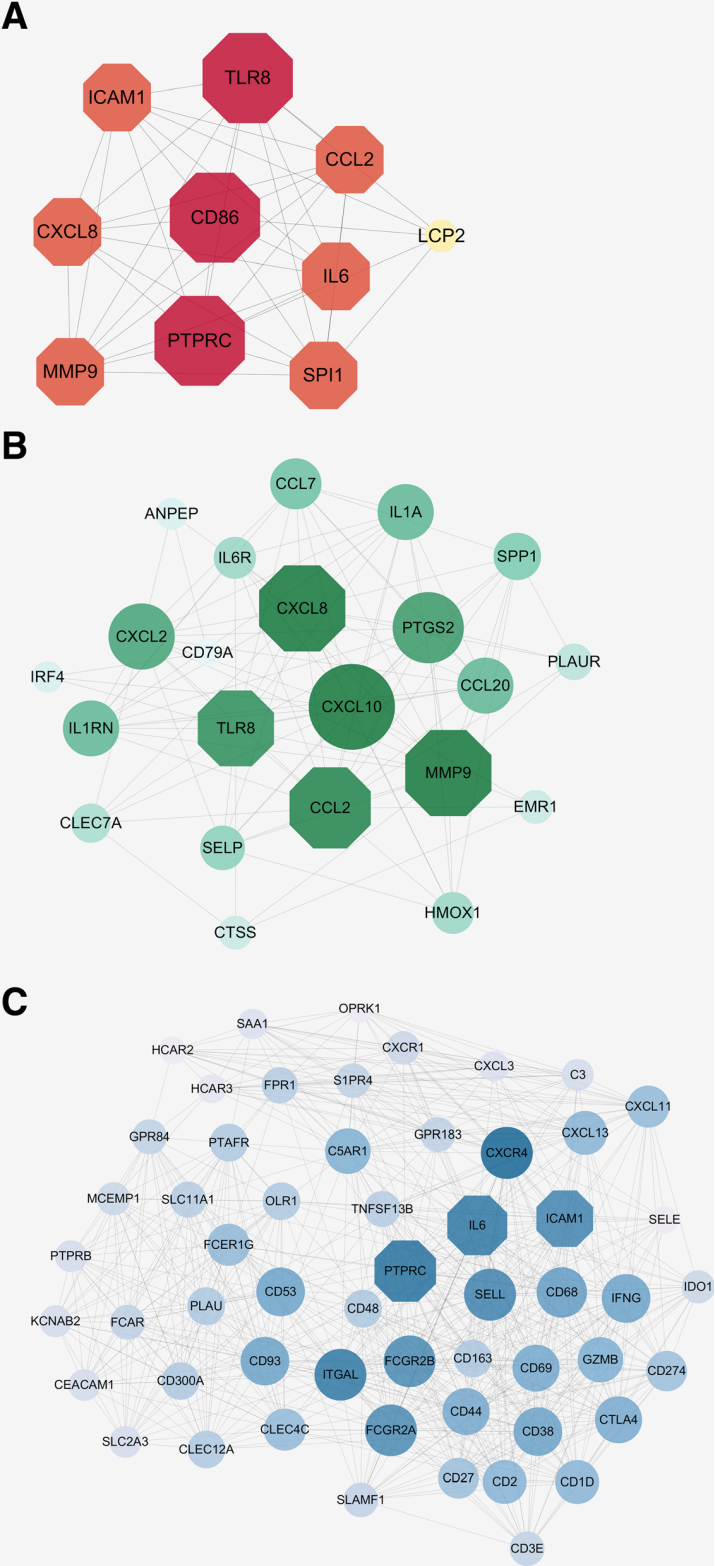
The hub genes and significant modules identified from the PPI network. **a** Top 10 hub mRNAs of pulpitis; **b** Module 1, with 22 nodes and 105 edges; **c** Module 2, with 56 nodes and 645 edges. The polygonal nodes represent the hub genes, and the round nodes are the other genes in the module network. The size and colour saturation of a node represent the degree of the node in each network

The PPI network constructed with the online STRING program consisted of 3873 edges and 465 nodes (Additional files 5 and 6). Used the MCODE plugin, two significant modules were obtained, one containing 22 nodes and 105 edges (module 1) and the other containing 56 nodes and 645 edges (module 2), as shown in Fig. 7. The hub genes of pulpitis from the PPI network are listed in Table 3.

**Table 3:** Top 10 hub genes with higher degrees of PPI network of pulpitis

Gene symbol	Description	Function	Degree
PTPRC	Receptor-type tyrosine-protein phosphatase C	Protein tyrosine-protein phosphatase required for T-cell activation through the antigen receptor. Acts as a positive regulator of T-cell coactivation upon binding to DPP4.	126
TLR8	Toll-like receptor 8	Key component of innate and adaptive immunity	100
CD86	T-lymphocyte activation antigen CD86	Receptor involved in the costimulatory signal essential for T-lymphocyte proliferation and interleukin-2 production, by binding CD28 or CTLA-4.	100
IL6	Interleukin-6	Cytokine with a wide variety of biological functions. Plays an essential role in the final differentiation of B-cells into Ig-secreting cells involved in lymphocyte and monocyte differentiation.	135
CCL2	C-C motif chemokine 2	Chemotactic factor that attracts monocytes and basophils	80
MMP9	Matrix metalloproteinase-9	May play an essential role in local proteolysis of the extracellular matrix and in leukocyte migration	83
IL8	Interleukin-8	A chemotactic factor that attracts neutrophils, basophils, and T-cells, major mediator of the inflammatory response	101
SPI1	Spi-1 Proto-Oncogene	This protein is a transcriptional activator that may be specifically involved in the differentiation or activation of macrophages or B- cells.	76
ICAM1	Intercellular adhesion molecule 1	Encodes a cell surface glycoprotein which is typically expressed on endothelial cells and cells of the immune system	83
LCP2	Lymphocyte cytosolic protein 2	Involved in T-cell antigen receptor mediated signalling	77

## Discussion

It is consistently challenging for clinicians to make accurate diagnoses regarding the level of dental pulp inflammation [4]. As new biological materials are developed, vital pulp therapies are increasingly used, which can increase the fracture resistance and long-time survival of teeth [31]. However, there is currently no accurate diagnostic tool for guiding dental pulp treatment; this shortcoming is an important cause of failure in vital pulp therapy [32]. Since histological examinations show that the extent of inflammation is not correlated with clinical manifestation, traditional diagnostic approaches based on symptoms or electrical/thermal pulp tests, require improvement [9, 33].

During inflammation, cells in human dental pulp, including odontoblasts, macrophages, vascular endothelial cells, precursor cells and other cells that can activate the immune system, secrete large amounts of cytokines, chemokines and neuropeptides, which have been reported to play critical roles in inflammation [34, 35]. Sivakami et al. found that the levels of IL-6 and the cytokine interleukin-1β (IL-1β) were clearly increased in saliva during pulpal and periapical inflammation [36]. An analysis of cytokines in pupal blood revealed that the levels of IL-8 were significantly higher in irreversible pulpitis than in caries-exposed pulp [37]. Several cytokines have been reported to be candidate diagnostic markers of pulpal inflammation [23, 38]. However, to the best of our knowledge, no biomarkers with high degrees of accuracy have yet been used in clinical examination to diagnose early pulpitis.

Research involving microarray analysis is rapidly expanding due to the rapid development of transcriptomic studies, resulting in an increasing understanding of the biological mechanisms underlying oral diseases [39]. Through the integration of several similar datasets, we can generate comprehensive expression profiles to identify key genes that can serve as biomarkers of pulpitis diagnosis and prognosis.

In the current study, expression data of pulpitis tissue from the GEO database were integrated and then used to identify the underlying characteristics of DEGs and candidate biomarkers for diagnosing the inflammation level of dental pulp. Li et al. reported DEGs and enrichment results obtained by analysing data in the GSE77459 dataset [40]. In contrast, we extracted data from GSE77459 and GSE92681 and integrated the mRNA expression data to remove batch effects and improve the quality of the data. Batch effects are the effects of technical differences unrelated to biological variation that are caused by the processing and measurement of samples in different batches, such as experiments that are conducted at different times or with different methods by different technicians [41]. The ComBat function and sva function of the SVA package that we used for data processing are useful for removing both known batch effects and other potential latent sources of variation [28]. After data processing, GO and KEGG pathway analyses were performed. The enrichment results revealed that the identified DEGs are involved in processes associated with dental pulp inflammation. Ten hub genes of pulpitis (PTPRC, TLR8, CD86, IL6, CCL2, MMP9, CXCL8, SPI1, ICAM1 and LCP2) were identified from the PPI network, and the possible mechanisms by which hub genes induce inflammation were investigated by GSEA and found to involve the complement system, IL6/JAK/STAT3 signalling pathway and inflammatory response pathways. Enriched KEGG pathways identified in both the present study and that of Li et al. [40] were cytokine-cytokine receptor interaction and chemokine signalling pathway, and hub genes common to the two studies were IL6, CXCL8, PTPRC, CCL2 and ICAM1.

PTPRC encodes protein tyrosine phosphatase (PTP), a signalling molecule that regulates various kinds of cellular processes and plays a critical role in the immune system. PTPRC can negatively regulate cytokine receptor signalling by suppressing the JAK signalling pathway [42]. PTPRC is expressed at low levels in normal dental pulp tissue [43] but at high levels in pulpitis tissue, as described above. CD86 is a receptor that commonly participates in T-lymphocyte proliferation and IL-2 production, acting as a negative regulator for the immune system [44]. Miyuki Azuma et al. studied immune responses in mouse dental pulp and found that expression of CD86 was enhanced in dental pulp after cusp trimming but disappeared within 2 h, with CD86 migrating into the regional lymph nodes at 24 h after acid treatment [45]. Chemokines activate and support the process of dental pulp inflammation. Accordingly, increased expression of CCL2 has been observed in chronic periapical lesions, indicating an association between chemokines and dental pulp inflammation [46, 47]. In addition, IL6, MMP9, TLR8, CXCL8, and ICAM1 have been reported to be associated with immunity and inflammation in dental pulp [1, 17, 48–51]. The above hub genes may play critical roles in pulp inflammation and therefore be potential biomarkers for use in pulpitis diagnosis. However, associations of LCP2 and SPI1 with pulpitis have not been reported previously.

During the treatment of deep caries and traumatic exposure of dental pulp, it is essential to assess whether the pulp inflammation is reversible. It has been reported that inflamed pulp tissue might produce some biomarkers that are secrete to the external environment [13, 14], making tests of dental pulp blood and dental fluid in pulpitis possible. Johannes et al. [17] used heparinized 10-mL microcapillary tubes to collect pulp blood samples when the dental pulp was exposed during caries removal. The MMP9 levels from blood sample of irreversible pulpitis were highly increased compared with those from blood samples of asymptomatic or reversible pulpitis teeth. In addition, dental fluid contained within dentinal tubules has been proposed to be potentially useful as a biomarker of different stages of pulpitis. By detecting the dental fluid in tooth cavities using polyvinylidene difluoride membrane, Brizuela et al. [18] found that the biomarkers of fibroblast growth factors acid (FGF-acid), interleukin-1α (IL-1α), IL-6, and tissue inhibitor of metalloproteinases 1 (TIMP-1) may collectively be useful for molecular diagnostics in pulpitis. Due to its advantages of ease-of-use and non-invasiveness, the dental fluid test is a promising method for the molecular diagnosis of pulpitis. However, dental pulp blood analysis might more accurately reflect the pathophysiologic conditions of dental pulp in inflammation [52]. The goal is to develop a low-cost, non-invasive, chair-side rapid method of pulpitis diagnosis. It has been reported that a rapid chair-side test of MMP8 in gingival crevicular fluid can be used to distinguish periodontitis from gingivitis and healthy gingiva [53].

However, in addition to the challenges of sample collection, many difficulties in the analysis and application of biomarkers need to be overcome. Much progress remains to be made in developing an effective method for molecular diagnosis in the clinical setting, which is the one of the limitations of the current study. In addition, due to the paucity of available datasets of pulpitis in the GEO database, the sample size in this study was limited. We will increase the sample size in a future study if additional datasets can be retrieved from the database.

## Conclusions

In summary, the present study analysed merged datasets of pulpitis tissue, aiming to gain insight into methods for the diagnosis or treatment of different inflammatory levels of pulpitis. In addition to investigating the possible regulatory mechanisms of DEGs, we screened key genes as biomarker candidates for the diagnosis of pulpitis, including PTPRC, CD86, CCL2, IL6, TLR8, MMP9, CXCL8 and ICAM1. The use of bioinformatic methods to analyse merged datasets is effective for identifying biomarkers of diseases. The present findings require further validation in future studies.

## Supplementary information

**Additional file 1.** Details of expression matrix from integrated datasets.

**Additional file 2.** Details of differentially expressed genes of pulpitis.

**Additional file 3.** Results of GO enrichment analysis of DEGs.

**Additional file 4.** Results of KEGG pathway analysis of DEGs.

**Additional file 5.** Results of STRING analysis of DEGs.

**Additional file 6.** Details of PPI network.

## Data Availability

The datasets generated and analysed during the current study are available in GEO DataSets repository, https://www.ncbi.nlm.nih.gov/gds.

## Abbreviations

GEO: Gene Expression Omnibus
DEGs: Differentially expressed genes
GO: Gene Ontology
KEGG: Kyoto Encyclopedia of Genes and Genomes
GSEA: Gene Set Enrichment Analysis
PPI: Protein–protein interaction
STRING: Search Tool for the Retrieval of Interacting Genes/Proteins
AAE: American Association of Endodontists
MMP: Matrix metalloproteinase
CXCL8: Interleukin-8
TIMP: Tissue inhibitor of metalloproteinases
limma: Linear Models for Microarray data
adj. P. val: Adjusted *p*-value
FC: Fold change
BP: Biological process
MF: Molecular function
CC: Cellular component
DAVID: Database for Annotation Visualization and Integrated Discovery
FDR: False discovery rate
NES: Normalized Enrichment Score
PCA: Principal component analysis
IL6: Interleukin-6
PTPRC: Receptor-type tyrosine-protein phosphatase C
TLR8: Toll-like receptor 8
CD86: Lymphocyte activation antigen CD86
CXCL8: Interleukin-8
ICAM1: Intercellular adhesion molecule 1
MMP9: Matrix metalloproteinase-9
CCL2: C-C chemokine receptor type 2
LCP2: Lymphocyte cytosolic protein 2
SPI1: Transcription factor PU.1
IL-1β: Cytokines interleukin-1β
