# Supplementary figures and images for: Diagnostic biomarker candidates for pulpitis revealed by bioinformatics analysis of merged microarray gene expression datasets
Source: BMC Oral Health. 2020 Oct 12;20:279. doi: 10.1186/s12903-020-01266-5 (PMC7552454; doi:10.1186/s12903-020-01266-5)

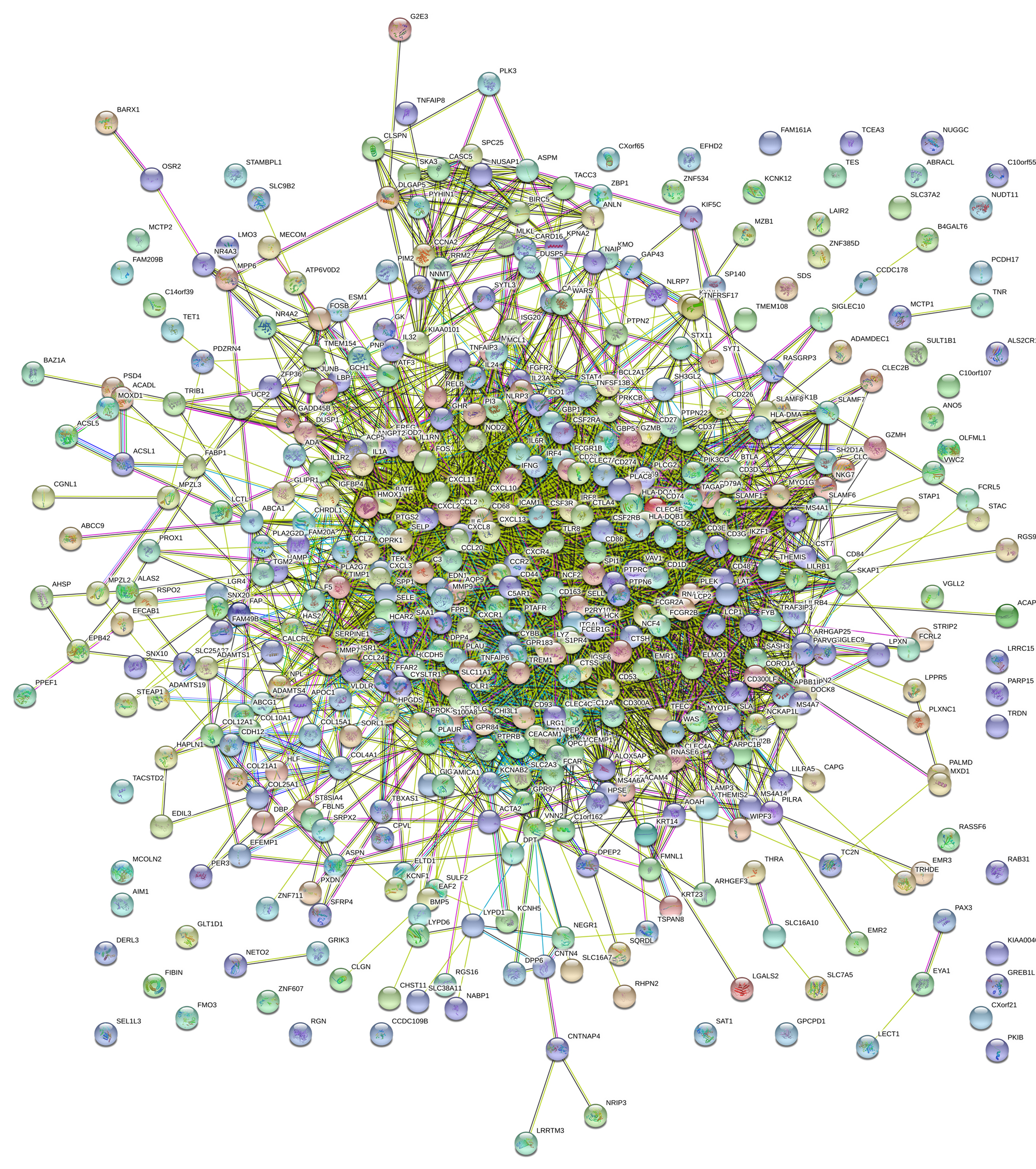

Supplement: Supplementary file 6 — Additional file 6. Details of PPI network. [file 12903_2020_1266_MOESM6_ESM.jpg]
